# Effects of age simulation suits on psychological and physical outcomes: a systematic review

**DOI:** 10.1007/s10433-022-00722-1

**Published:** 2022-09-22

**Authors:** Thomas H. Gerhardy, Anna Schlomann, Hans-Werner Wahl, Laura I. Schmidt

**Affiliations:** 1 Institute of Psychology, Heidelberg University, Hauptstr. 47-51, 69117 Heidelberg, Germany; 2 Network Aging Research, Heidelberg University, Bergheimerstr. 20, 69115 Heidelberg, Germany; 3 Institute for Educational Science, Heidelberg University of Education, Keplerstr. 87, 69120 Heidelberg, Germany

**Keywords:** Attitude, Empathy, Ageing simulation, Geriatric education, Physical ageing

## Abstract

Age simulation suits (ASS) are widely used to simulate sensory and physical restrictions that typically occur as people age. This review has two objectives: first, we synthesize the current research on ASS in terms of the observed psychological and physical effects associated with ASS. Second, we analyze indicators able to estimate the validity of ASS in simulating “true” ageing processes. Following the PRISMA guidelines, eight electronic databases were searched (BASE, Cinhal, Cochrane, Google Scholar, ProQuest, PsychINFO, Pubmed, and Web of Science). Qualitative and quantitative studies addressing effects of ASS interventions regarding psychological outcomes (i.e., empathy, attitudes) or physical parameters (i.e., gait, balance) were included. The Mixed Methods Appraisal Tool was applied for quality assessment. Of 1890 identified citations, we included 94 for full-text screening and finally 26 studies were examined. Publication years ranged from 2001 to 2021. Study populations were predominantly based on students in health-related disciplines. Results suggest that ASS can initiate positive effects on attitudes toward (*d*_weighted_ = 0.33) and empathy for older adults (*d*_weighted_ = 0.54). Physical performance was significantly reduced; however, there is only little evidence of a realistic simulation of typical ageing processes. Although positive effects of ASS are supported to some extent, more diverse study populations and high-quality controlled designs are needed. Further, validation studies examining whether the simulation indeed reflects “real” ageing are needed and should build on reference data generated by standardized geriatric assessments or adequate comparison groups of older adults.

*Prospero registration*: 232686.

**Supplementary Information:**

The online version contains supplementary material available at 10.1007/s10433-022-00722-1.

## Introduction

The application of age simulation suits (ASS) has been undergoing continuous development since the 1990s, when the automotive industry started to use the first prototypes. ASS were originally constructed to raise engineers’ awareness of age-related and differently caused physical impairments when designing new cars. Concurrently in the gerontological arena, educational programs involving ASS emerged with the ambition to reduce negative attitudes toward older adults in caregiving settings (Galanos et al. 1993; Pacala et al. 1995), enhance empathy and role-taking in relation to older adults, and explore the benefit of experience-based education at large. In this paper, ASS are defined as devices that simulate both physical and sensory restrictions by using additional weights, hearing protection and specifically designed goggles.

Framing the use of ASS more generally, the global transformation toward an increasingly proportion of older population brings along challenges and requirements for the health care system, i.e., understanding what ageing means on various levels. More specifically, ASS come with the ambition to foster processes of empathy and role taking with respect to understanding daily life dominated by physical and sensory impairments. The main expectation here is the increase in positive attitudes toward older adults through age simulation (Bennett et al. 2016; Chen et al. 2015). Primary target groups are health care personnel, family caregivers, and younger age groups in general (Bowden et al. 2020). ASS meanwhile offer many different means to simulate impairments in gross and fine-tuned motor behavior, hearing, and vision that show strong associations with older age and can be seen as markers of ageing (Bergman and Rosenhall 2001). For example, various versions of goggles and hearing protectors mimic different magnitudes of impaired vision or hearing, additional weights (vest, ankle and wrist cuffs) simulate reduced stamina and physical capacity, while joint bandages are used to limit the range of motion (Allen 2018; Lauenroth et al. 2017; Scherf 2014).

Since a growing popularity can be observed regarding ASS in different settings in the recent years, it is fundamentally important to thoroughly and empirically investigate the possibilities and limitations of such simulations. On the one hand, no false picture of age-related limitations should be conveyed, which may establish fears and concerns about later life or regarding views on ageing and older adults. On the other hand, there is a lack of research that explores if ASS allow a realistic simulation of ageing processes and if the simulated age-range corresponds to average functional abilities of older adults in third (60–79 years) or fourth age (80+). So far, predominantly young participants were included in ASS studies and there is some evidence that the simulated impairments did not correspond to old age but rather middle-adulthood. A realistic simulation (i.e., reaching the average functional impairments of a 70, 80, or 90 years-old person) would also be of importance if ASS are used in the development phases of geriatric assistive devices, when the risk of falling is still high and older adults cannot be consulted for first pilot studies because of practical or ethical reasons. Although there is a relatively large body of case-like reports often containing positive experiences with the application of ASS, a comprehensive systematic overview of the currently existing research on the psychological and physical outcomes of wearing an ASS is missing. A scoping review on age simulation interventions was published in 2017, but included only two studies, which were conducted among nursing students (Coelho et al. 2017). A second review article focused on the effects of ASS on attitudes, empathy and anxiety levels among student populations only (Eost-Telling et al. 2020). A third most recent review focused on the educational effects of ASS on person-centered care (Bowden et al. 2021).

These reviews of existing data on ASS have some shortcomings and gaps in their syntheses. First, none of the available reviews addressed outcomes related to physical functioning such as strength loss, gait parameters or balance issues. Second, none quantified and discussed the validity of simulated physical impairments as a realistic simulation in comparison with “real ageing.” Third, existing reviews show limitations on included study populations. Eost-Telling et al. (2020) and Bowden et al. (2021) only included studies with participants in the healthcare sector, which limits generalizability. Fourth, they also included geriatric (medication) games, using role-playing, i.e., with focus on medication intake, meaning that some participants only acted as observers so that not everybody experienced the simulation first-hand. Further, game-based approaches did not apply a complete ASS, but typically used certain parts of the ASS set-up, thus not allowing for a full and more holistic experience.

Therefore, the first objective of the present review was to synthesize the current research examining the effect of ASS interventions on psychological as well as physical outcomes. Psychological outcomes have partly been addressed by Eost-Telling et al. (2020) and Bowden et al. (2021), but several more recent studies have not been included in their synthesis yet. In addition, we did not exclusively focus on students from health professions like previous reviews, but also include studies targeting general populations of younger and middle-aged adults.

Our second objective was to analyze indicators able to estimate the validity of existing ASS in simulating typical ageing processes, i.e., by drawing on reference values of established assessments or via comparisons with the performance of older adults in the target age of the simulation.

## Methods

We checked PROSPERO (https://www.crd.york.ac.uk/prospero/) for similar systematic reviews on this topic or ongoing projects. No registered review could be found. The systematic review was prospectively registered in PROSPERO (CRD42021232686, February 28, 2021) and was conducted in accordance with the PRISMA statements (Moher et al. 2009).

### Search strategy

In June 2020 a literature search was conducted in seven electronic databases (BASE, Cinhal, Cochrane, ProQuest, PsychINFO, Pubmed, and Web of Science) without time limits for publication years. Search terms and combinations were customized for each database as shown in the supplemental material, Table 1. In a second step, Google Scholar was used to find additional relevant studies including gray literature. Further articles were added following manual reference search and an update scan for new publications in September 2021.

**Table 1:** Characteristics of included studies

Author	Country	Study design	Number of participants	Sample characteristics	% of males	Age ± SD (years)	Age range (years)	Simulation program	Duration of intervention (min)	Assessment	Main results
Akpinar Söylemez et al. (2021)	Turkey	Non-randomized quantitative	92	Nurses	15.2	35.67 ± 8.37	n/a	GERT ASS lecture about age and ageing day 1; daily activities day 2	30-40	pre/post KAOP	Simulation-based training program can increase attitudes toward older adults
Allen (2018)	United States	Non-randomized quantitative	59	Healthy, no cardiovascular disease or orthopedic limitations	30.5	n/a	20-24	GERT ASS geriatric assessments: SFT, SPPB, gripstrength	10-15	ASD questionnaire	No sig. difference in attitudes toward older adults
Bowden et al. (2020)	Australia	Qualitative	15	Nurses, -students, -assistants	33.3	n/a	18-64	GERT ASS daily hospital activities	7-15	Debrief discussions; follow-up focus groups	ASS program is beneficial educational approach and enhance the insight into the ageing process
Cheng et al. (2020)	Hong Kong	Randomized quantitative	139 IG: 69 CG: 70	Nursing students and health professions	24.5	21.2 ± 3.5	18-29	CG: placebo clothes IG: Koken ASS Senior Simulation Program: Reading fill out a form, sort medications, eating, listening to audio, sitting in a chair, walking, smell and taste food	30	C-KAOP WCOP C-FAQ (pre-only)	No difference in WCOP between IG & CG, but increased C-KAOP in both groups
Filz (2010)	Germany	Non-randomized quantitative	253 IG: 128 CG: 125	Medical students	IG: 37.5 CG: 36.8	IG: 25.57 ± 3.82 CG:25.39 ± 3.65	n/a	Self-made ASS up/down stairs, walking, make a bread, lace up shoes, take a pill	20	Self-developed questionnaire “understanding for older people”	Improved understanding and empathy toward older adults after the intervention IG and CG
Hsu et al. (2016)	Taiwan	Non-randomized quantitative	134	Nurses	1.5	n/a	21-30 (*n* = 91) ≥ 31 (*n* = 43)	self-made ASS ageing simulation intervention program “Walked and climbed” daily activities	10-15	Pre/3 months post Attitudes Toward the Older People Scale, WCOP	Increased WCOP after the intervention no sig. change in attitudes toward older adults
Jeong et al. (2017)	South Korea	Mixed methods	70	Nursing students	17.1	20.4 ± 4.03	19-44	Sakamoto ASS “Senior Simulation Program” (SSP) orientation program daily activities; sitting, reading, walking, eating	13	ASD pre-/post-follow-up-test, qualitative written review (+ interview 9 students)	ASD became negative after the SSP (post), but more positive after sharing the feelings (follow-up)
Jeong and Kwon (2020)	South Korea	Non-randomized quantitative	65	Nursing students	7.7	19.5 ± 0.89	19-22	Sakamoto ASS “Aging Suit Experience Program”; lying/sitting down on/getting up from a bed/chair, walking, up/down stairs, reading, hearing, open a bag, drink	n/a	Semantic Differential, behavior toward elderly scale	Subjects’ behavior toward older adults improved, maintained for 3 months after the program
Lauenroth et al. (2017)	Germany	Non-randomized quantitative	178	Healthy individuals	32.0	50.4 ± 16.4	18-85	GERT ASS gait analysis	45	Gait performance in four groups with ASS, two groups without ASS	Gait performance of young corresponds to performance of older group
Lavallière et al. (2017)	United States	Mixed methods	22	Healthy individuals	22.2	24.0 ± 2.57	20-29	AGNES ASS assessments for physical performance	Up to 120	Postural balance, neck/shoulder range of motion, lower back/hamstring flexibility, gait analysis	Majority experienced a decline in performance with ASS
Lee and Teh (2020)	Malaysia	Mixed methods	133 IG: 52 CG: 68	Pharmacy students	22.5 20.6 25.0	19.5 ± 0.8 19.5 ± 0.7 19.5 ± 0.8	n/a	CG: lecture IG: lecture + Nagoya ASS fill out a form, walking, stand up/sit down on a sofa/chair, comb hair, pick up sth. from the floor	10	JSE-HPS, open-end questions	Self-rated knowledge and understanding on the physical limitations of ageing were similar between both groups
Losa Iglesias et al. (2020)	Spain	Non-randomized quantitative	54	Nursing students	22.7%	21 ± 1.42	20.61-21.38	GERT ASS daily activities; up/down stairs, sit down/get up from a chair, putting on shoes	60	JSE-HPS, TMMS-24, PANAS, open-end questions	Increased empathy in nursing students after the simulation experience
Lucchetti et al. (2017)	Brazil	Non-randomized quantitative	230 IG1: 72 IG2: 82 CG: 76	Medical students	IG1: 34.6 IG2: 60.5 CG: 47.2	IG1:18.71 ± 1.43 IG2: 19.7 ± 2.72 CG:19.91 ± 3.07	19-39	CG: control group IG1: “Aging Game” age simulation tools (with weights); walking around obstacles IG2: “Myths of Aging”	25	UCLA, Palmore FAQ-1, MMSS	IG1: increased empathy, worsening attitudes IG2: improved attitudes, no change in empathy
Mandegari Bamakan et al. (2021)	Iran	Non-randomized quantitative	70 IG: 35 CG: 35	Nursing students	IG: 42.9 CG: 55.7	IG: 20.8 ± 1.16 CG:21.25 ± 2.20	20-26	CG: lecture IG: lecture + Unknown ASS simulation experience walking, open door, up/down stairs, calling, eat and drink	120	FAQ, KAOP	Improved knowledge and positive attitudes toward older adults
Mohamed et al. (2017)	Egypt	Randomized quantitative	82 IG: 41 CG: 41	Nursing students	19,8 IG: 22 CG: 17.1	21.50 ± 0.5 IG: 21.6 ± 0.5 CG: 21.5 ± 0.6	21-23	CG: lectures Self-made ASS IG: lectures + simulation game: eating, drinking, chose coloured pins, listening instructions, walking with walker/wheelchair, open doors, wash hands, open jar, button up coat	180	KAOP, Structured interview, Experience with Ageing	Ageing simulation improves knowledge, awareness about ageing and understanding for their problems
Perot et al. (2020)	France	Quantitative descriptive	306	Healthcare professionals	31	42	18-68	GERT ASS up/down stairs, lying down/getting up, sitting, drinking, eating	15	Self-developed questionnaire on difficulties	Improved participants’ opinions on difficulties experienced by older people
Robinson and Rosher (2001)	United States	Quantitative descriptive	49	Third-year medical students	n/a	n/a	n/a	Self-made ASS lecture + simulation experience: reading, sort coloured paper, prepare medication, eating/tasting, lace up shoes, fill out form, count out change	180	ASD	Improvement in attitudes toward ageing
Ross et al. (2013)	United Kingdom	Mixed-methods	86	Healthcare assistants/professional, nurses	n/a	n/a	n/a	ASS role playing, being a patient	60	IMTEE + semi-structured interviews	Improved confidence
Rueffert and Bullinger (2020)	Germany	Non-randomized quantitative	330 IG: 197 CG: 133	In- and outpatients’ staff of geriatric care	15.4	36.2 ± 14.2	17-63	CG: observer IG: MAX ASS stairs up/down, sitting down/getting up, combing hair, go to the toilet/bed, open a medicine box	120	Self-developed-questionnaire about subjective competence in relation to older people and sensitization to ageing after the training	Higher sensitization post intervention in IG versus CG
Sari et al. (2020)	Turkey	Mixed methods	Phase 1: 260 Phase 2: 30 3 groups á 10	Nursing students	50	22.46 ± 1.72	n/a	Sakamoto ASS phase 2 group: up/down stairs, walking, go to a market, shopping, reading	n/a	KAOP, BES; semi-structured interviews	KAOPS & BES improved after phase 2
Scherf (2014)	Germany	Non-randomized quantitative	38 young: 28 old: 10	Technical staff (automotive industry)	38	25.04 ± 3.14 52.70 ± 3.42	20-31 47-58	MAX ASS assembly tasks (engineering)	180	Subjective load, heart rate, time to complete a task, joint flexibility	Time to complete the tasks increased in young participants with ASS and was comparable to older participants without ASS
Varkey et al. (2006)	United States	Non-randomized quantitative	84	Medical students	45.2	n/a	20-25 (78.3%) 26-30 (14.5%) 30-35 (6.0%) > 36 (1.2%)	Self-made ASS “Modified Aging Game” grocery shopping, being fed sitting in a wheelchair	180	MMS, ASD	Improved attitudes and empathy toward older adults
Vieweg and Schaefer (2020)	Germany	Non-randomized quantitative	20	Healthy young adults	50	22.3	20-28	GERT ASS geriatric motor and cognitive assessments	75	PEPS, physical state, MS; SFT, strength, flexibility, aerobic endurance, TUG, DS, PPT	The performance decreased while wearing the ASS
Watkins et al. (2021)	United Kingdom	Non-randomized quantitative	30	Healthy young adults	53.3	n/a	20-40	The Adam, Rouilly AK060 ASS Geriatric assessments	n/a	FRT, TUG, BBS	The ASS decreased the performance
Yu and Chen (2012)	Taiwan	Non-randomized quantitative	83 IG: 43 CG: 40	Nursing home staff	1.2	48.0 ± 8.8 50.4 ± 6.6 54.4 ± 10.2	n/a	CG: nothing IG: self-made ASS; Elderly Simulation Program Sitting in a wheelchair, filling out forms, eating, go to the bathroom, reading, up/down stairs, get up from a bed	20	Pre/post questionnaires “Knowledge and attitudes toward older adults + motivation to care for older adults”	IG: improved knowledge about ageing and attitudes toward older adults
Zijlstra et al. (2016)	Netherlands	Non-randomized quantitative	75	Bachelor students- facility management	33	20.0 ± 1.8	n/a	Gero ASS wayfinding tasks	n/a	Route efficiency and time, heart rate, respiratory rate, energy expenditure	With ASS worse in wayfinding, slower walking, sig. higher heart/respiratory rate

*n/a* not applicable, *SD* Standard Deviation, *ASS* Age Simulation Suit, *CG* Control Group, *IG* Intervention Group, *ASD* Aging Semantic Differential, *BES* Basic Empathy Scale, *JFE-HPS* Jefferson Scale of Empathy-Health Professions Students, *IMTEE* Integrated Model of Training Evaluation and Effectiveness, *KAOPS* Kogan’s Attitudes Toward Older People Scale, *MSS* Maxwell Sullivan Scale, *Palmore FAQ-1* Palmore Facts About Aging Quiz, *UCLA-GA* UCLA Geriatric Attitudes Test, *WCOP* Willingness To Care For Older People Scale

### Eligibility criteria

Studies were included if they (a) applied ASS to mimic physical and sensory limitations; (b) reported qualitative, quantitative, or mixed-methods outcomes regarding attitudes, understanding or empathy toward older adults and/or assessments of physical functioning (i.e., gait, mobility, balance, strength); and (c) if they were published in English or German language.

We also included gray literature and excluded reviews, meta-analyses, comments, protocols, case reports and conference papers/presentations. Studies simulating specific medical conditions (i.e., hemiparesis) were excluded, as we focused on typical and frequent ageing-related physical and sensory limitations. Educational board games or role-plays, which concentrated on single sensory or physical restrictions and did not explicitly report an intervention for all participants, were excluded. Studies which did not report any results or did not initially aim to study effects with a clear research question (i.e., evaluations of seminars) were excluded as well.

### Selection

We screened all articles by title and abstract to identify potentially relevant manuscripts based on the inclusion criteria. At this level, only very obviously ineligible titles were removed. For the full-text screening, two authors (AS, LS) independently assessed 50% of the potentially eligible articles while one author (TG) independently assessed all. Disagreements were resolved through discussion and involvement of the respective uninvolved author (AS or LS). Subsequently, the first author extracted information on the study (author, title, year of publication, country of origin), study characteristics (design, methods, sample size, types and modalities of the simulation and duration of interventions), participants’ characteristics (age, gender), and indices regarding self-reported psychological outcomes (i.e., empathy) and/or physical performance outcomes (i.e., gait, flexibility). If relevant data were not available, we contacted the authors of the study to request missing information.

### Quality assessment and statistical analyses

To assess the quality of selected articles the Mixed Methods Appraisal Tool (MMAT) for systematic mixed methods reviews was used (Hong et al. 2019). Two authors (AS, LS) independently assessed 50% of the articles, while one author (TG) assessed all articles. Disagreements were resolved by discussion, with the involvement of respective uninvolved author (AS or LS) if needed. To compare the effects of ASS interventions between studies, we calculated pre-to-post effect sizes (Cohen’s *d*) from the indices reported or received on request (Lenhard and Lenhard 2017). Cohen’s *d* is interpreted as followed: no effect: *d* = 0-0.1; small effect: *d* = 0.2-0.4; medium effect: *d* = 0.5-0.7; large effect: *d* ≥ 0.8 (Cohen 1988). Subsequently, we calculated pre-to-post weighted mean effect sizes for attitudes and empathy separately by weighting each effect size by the respective sample size of the study participants receiving an ASS intervention. Those weighted means also include pre-to-post differences of the intervention groups of the few (randomized) controlled studies, weighted by the number of participants in the respective intervention group. For the latter designs, we additionally calculated effects sizes for group differences (control group vs. intervention group), taking into account baseline scores (Morris 2008).

## Results

**Fig. 1:**
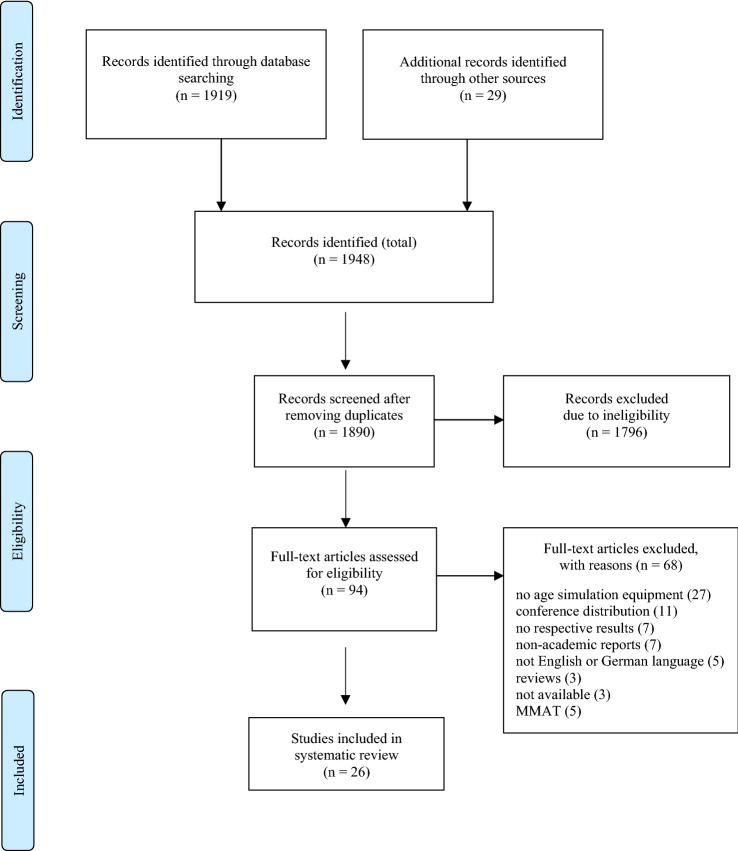
PRISMA flow diagram. *Note* MMAT = Mixed Methods Appraisal Tool for quality assessment

Figure 1 illustrates the results of the screening process according to the PRISMA guidelines (Moher et al. 2009). A total number of 1948 articles was found. 1890 abstracts were screened after removing duplicates and 94 were included for the full-text screening. At full-text level, 68 studies were excluded because of the following reasons: geriatric medication/ageing games, role plays or similar studies not using ASS (*n* = 27), conference contributions (*n* = 11), not reporting respective results (*n* = 7), non-academic reports (i.e., newsletter) (*n* = 7), language not English or German (*n* = 5), review articles (*n* = 3), unavailable after contacting the authors (*n* = 3). After the quality assessment, further studies were excluded due to insufficient data to answer the two screening questions (see next section; *n* = 5). Finally, 26 articles were included in the synthesis. Of those, 15 studies had not been included in previous review articles.

### Quality assessment

The MMAT (Hong et al. 2019) for quality assessment offers the opportunity to evaluate diverse study designs in five categories (1) qualitative, (2) quantitative randomized, (3) quantitative non-randomized, (4) quantitative descriptive, and (5) mixed methods. The tool draws on two screening questions. (1) “Is there a clear research question?” (2) “Do the collected data allow to address the research question?” and five additional quality criteria, varying depending on the category of study design. Results of quality assessment revealed a heterogeneous picture of study quality. Two studies that did not meet the first screening question and three studies that did not meet the second screening question of the MMAT analysis and were therefore excluded from the following synthesis. Three studies announced written or oral feedback in seminars as qualitative results, but used quantitative descriptive methods to analyze data and were therefore evaluated in the respective category of the MMAT. With respect to study design, we included qualitative studies (*n* = 1), quantitative randomized (*n* = 2), quantitative non-randomized designs (*n* = 16), quantitative descriptive designs (*n* = 2) and mixed methods studies (*n* = 5). One of the randomized trials also included qualitative results and was therefore assigned to the mixed methods category.

Results of the MMAT indicated that eight articles met all five quality criteria of the respective design, twelve articles did not meet one criterion and six did not meet two criteria. More specifically, the qualitative study met all relevant criteria, the two quantitative randomized studies received good ratings, with the exception that assessors’ blinding was unclear (*n* = 1) or not implemented (*n* = 1). Among the sixteen quantitative non-randomized studies, eight studies did not describe nor analyze confounders, while two studies included participants that were not suitable or representative for their target population. For the two quantitative descriptive studies, it remained unclear if authors controlled for nonresponse bias in both studies and in one study, participants were not suitable or representative. Among the five mixed methods studies, there was one study missing an explanation for integrating qualitative and quantitative methods and one study missing a link between chosen methods and their interpretation. Furthermore, two studies lacked an explanation for divergences between quantitative and qualitative results (*n* = 2).

### Study characteristics

Key characteristics of the included studies are presented in Table 1. Studies were mostly conducted in Europe (*n* = 11), followed by Asia (*n* = 7), the United States (*n* = 3), Turkey (*n* = 2) and one from Australia, Egypt and Iran, respectively. Publication dates ranged from 2001 to 2021, with twelve of the 26 articles published in 2020 and 2021. Sample sizes varied depending on research method and design used. The nineteen studies collecting quantitative data with questionnaires reported the largest numbers of participants (range: *N* = 49-330), followed by studies on physical performance measurements (range: *N* = 20-178), and qualitative methods (range: *N* = 15-64). The majority of studies (*n* = 21) predominantly included participants between 20 and 30 years, due to the fact that most studies were conducted with pharmacy, medicine or nursing students, and younger health care staff. The duration of the procedures including the application of the ASS, habituation phase (if implemented) and the execution of a diverse range of tasks under ASS conditions ranged from 10 min (Hsu et al. 2016) to 4 h (Bowden et al. 2020). Some studies were embedded in university courses, consisting of an introduction by means of a lecture (Akpinar Söylemez et al. 2021; Jeong and Kwon 2020; Mohamed et al. 2017; Robinson and Rosher 2001; Yu and Chen 2012); others followed a workshop format (Filz 2010) including interactions with older adults (Lee and Teh 2020). In order to study mid- to long-term effects, follow-up designs were only used in three studies, but solely regarding psychological outcomes (Jeong et al. 2017; Jeong and Kwon 2020; Lee and Teh 2020), varying between 3 weeks and 3 months. The vast majority of studies (*n* = 20) aimed to find starting points to enhance the quality of care, and therefore addressed empathy, attitudes, and/or understanding as these are assumed to be critical skills for health professions. These outcomes were measured by questionnaires, qualitative interviews, or evaluations of group discussions. Another six studies tried to fathom if ASS can simulate diverse age-related impairments and used quantitative performance measurements, e.g., heart rate to determine the physical load, geriatric assessments, gait analysis, and cognitive tasks in one study.

### Findings on research question 1: effects of wearing an age simulation suit

#### Psychological outcomes

Detailed information on study results and calculated effect sizes can be found in Tables 2 and 3. Nineteen studies measured the effects of ASS on psychological outcomes quantitatively with established or self-developed questionnaires. The following instruments were applied (alphabetical order with frequency used in brackets): Aging Semantic Differential (ASD) (3), Attitude Toward the Older People Scale (1), Basic Empathy Scale (BES) (1), Integrated Model of Training Evaluation and Effectiveness (IMTEE) (1), Jefferson Scale of Empathy-Health Professions Students (JSE-HPS) (2), Kogan’s Attitudes Toward Older People Scale (KAOP) (5), Modified Maxwell-Sullivan attitudes toward the elderly Scale (MSS) (2), (Chinese-) Palmore’s Facts on Aging Quiz ((C)-FAQ) (3), Semantic Differential Scale (SD) (2), UCLA Geriatric Attitudes Test (UCLA-GA) (1), and Willingness to Care for Older People Scale (WCOP) (1). Four studies also used self-developed questionnaires.

**Table 2:** Results and calculated effect sizes of the included quantitative studies in controlled or between-subject designs

Author(s)	Intervention/control group (IG/CG)	Conducted questionnaire	T0 mean ± SD	T1 mean ± SD	*p* T0-T1	*p* T1 IG-CG	*d* T0-T1	*d* IG-CG	Findings
Cheng et al. (2020)	IG: Senior Simulation Program	KAOP	IG: 120.3 ± 12.3	IG: 124.8 ± 12.4	< .001	.374	0.36	0.07	↑ Increased attitudes toward older adults in IG and CG
	Koken ASS		CG: 122.8 ± 11.6	CG: 126.5 ± 10.0	< .001				
	CG: placebo clothing	WCOP	IG: 5.0 ± 1.3	IG: 5.5 ± 1.1	< .001				↑ Willingness to care for older adults in CG and IG
			CG: 5.4 ± 0.9	CG: 5.6 ± 10.0	< .001	.397	0.42	0.27	No sig. difference between groups
Filz (2010)	self-made ASSIG: “instant ageing program”	Attitudes score		IG: 40%	n/a	n/a			↑ Higher attitudes score in IG vs. CG
				CG: 29%					
	CG: lecture	Evaluation sheets		IG: 85%	n/a	n/a			↑ Higher empathy score in IG vs. CG
		Empathy score		CG: 57%					
Lee and Teh (2020)	IG: daily activities + workshop	JSE-HPS	IG: 111.5 ± 13.6	113.2 ± 14.5	.86	.81	0.12	0.04	No sig. change in empathy, felt back to baseline 3 months later in both groups
	Nagoya ASS		CG: 111.9 ± 11.3	113.1 ± 9.4			0.12		
	CG: workshop	Self-rated knowledge	n/a	n/a	n/a	.79			Self-rated knowledge and understanding on physical limitations post intervention similar between groups
		Empathy	n/a	n/a	n/a	.70			
		Support	n/a	n/a	n/a	.34			
Luchetti et al. (2017)	IG1: “Experiencing Aging”						- 0.36^a^		
	unkown ASS	UCLA-GA	IG1: 51.88 ± 4.67	IG1: 50.51 ± 5.19	.001	IG1-CG .809	- 0.28		↓ IG1 decreased attitudes
			IG2: 51.01 ± 5.25	IG2: 53.67 ± 4.16	< .001	IG2-CG < .001	0.56		↑ IG2 increased attitudes
				CG: 50.00 ± 5.68					
	IG2: “Myths about aging”	Palmore FAQ-1	IG1: 11.61 ± 2.74	IG1: 10.94 ± 2.28	.033	IG1-CG .104	- 0.27		↓ IG1 decreased knowledge
	no ASS		IG2: 11.13 ± 2.41	IG2: 17.58 ± 1.96	< .001	IG2-CG < .001	2.94		↑ IG2 increased knowledge
				CG: 10.22 ± 2.24					
	CG: no intervention	MSS attitudes	IG1: 15.53 ± 2.93	IG1: 16.78 ± 2.83	.007	IG1-CG .734	- 0.43		↓ IG1 decreased pos. attitudes
			IG2: 16.17 ± 3.65	IG2: 14.83 ± 3.46	< .001	IG2-CG .042	- 0.38		↓ IG2 increased pos. attitudes
				CG: 16.29 ± 4.2					
		MSS empathy	IG: 5.33 ± 1.62	IG1: 4.63 ± 1.31	.001	IG1-CG .124	0.48		↑ IG1 increased empathy
			IG2: 5.44 ± 2.37	IG2: 5.13 ± 2.10	.070	IG2-CG .824	- 0.21		IG2 no difference
				CG: 5.32 ± 2.09					
Mandegari and Bamakan et al. (2021)	IG: lecture about age & ageing, ageing simulation	Palmore FAQ-1	IG: 9.2 ± 2.6	IG: 15.3 ± 3.5	.001	.001	1.98	1.87	↑ Increased knowledge in IG post sig. difference between IG-CG post
	Unknown ASS		CG: 10.4 ± 2.9	CG: 11.3 ± 2.6	.30		0.33		
		KAOPS	IG: 114.69 ± 8.4	IG: 157.31 ± 10.7	< .05	.001	4.43	4.15	↑ IG increased positive attitudes post ↓ CG decreased positive attitudes post
	CG: lecture		CG: 113.34 ± 13.6	CG: 108.5 ± 16.6	< .05		- 0.32		
		Positive	IG: 58.06 ± 6.3	IG: 78.8 ± 6.9	.001	.001	3.14	0.53	↑ IG increased negative attitudes post ↓ CG decreased negative attitudes post
			CG: 56.9 ± 10.9	CG: 52.9 ± 8.4	.10		- 0.41		
		Negative	IG: 56.6 ± 6.2	IG: 78.5 ± 5.7	.001	.01	3.68	-0.82	
			CG: 56.3 ± 7.1	CG: 52.6 ± 10.1	.06		- 0.42		
Mohamed et al. (2017)	IG: lectures about age & ageing, simulation game	KAOPS					0.71^b^		
	Self-made ASS	Positive	IG: 53.7 ± 5.7	IG: 57.8 ± 7.0	.30	.20	0.64	0.53	No sig. difference between groups (post)
			CG: 54.7 ± 5.8	CG: 55.7 ± 7.8	.20		0.15		↑ Tendency to more positive attitudes in IG/CG post
	CG: lectures	Negative	IG: 49.8 ± 7.1	IG: 43.9 ± 8.1	< .001	.014	0.78	-0.82	↓ Decreased scores in negative attitudes in IG post
			CG: 48.2 ± 7.1	CG: 48.2 ± 7.3	1.0		0.00		
		Knowledge about normal ageing changes	IG: 13.3 ± .3.3	IG: 21.0 ± 2.6	< .001	< .001	2.59	1.23	Sig. difference between IG-CG
			CG: 13.0 ± 3.6	CG: 16.4 ± 3.5	< .001		0.96		↑ Increased knowledge in IG
Rueffert and Bullinger (2020)	IG: lectures about age & ageing, tasks of daily living	Self-developed: cognition		IG: 5.03 ± 0.73		.004			↑Increased empathy and understanding subjects wearing the ASS had higher scores in
	MAX ASS			CG: 4.76 ± 0.85					
	CG: lectures about age & ageing, observed the IG	Empathy and understanding		IG: 5.32 ± 0.76		.009			
				CG: 5.12 ± 0.80					
		Motivation		IG: 4.87 ± 0.80		.020			
				CG: 4.65 ± 0.86					
Yu et al. (2012)	IG: lectures about age & ageing, tasks of daily living	Nursing Assistants’ knowledge About Aging Scale	IG: 27.4 ± 5.5	IG: 33.3 ± 5.8	< .001		1.04	1.21	↑ Sig. increased knowledge for IG
	Self-made ASS		CG: 28.4 ± 4.8	CG: 28.0 ± 4.1	.61		- 0.09		
	CG: lectures about age & ageing	NA Attitudes toward Older Adults	IG: 62.4 ± 6.1	IG: 65.2 ± 6.8	.001		0.43	0.46	↑ Sig. increased attitudes toward older adults in IG
			CG: 65.3 ± 5.6	CG: 65.4 ± 8.8	.95		- 0.01		
		NA Motivation to Care	IG: 46.6 ± 4.5	IG: 47.7 ± 4.2	.06		0.25		No sig. change in motivation to care in IG and CG
			CG: 48.3 ± 4.6	CG: 47.1 ± 4.6	.12		- 0.26		

n, numbers; s, seconds; cm, centimeters; °, degrees; ASS, Age Simulation Suit; CG, Control Group; IG, Intervention Group; ASD, Aging Semantic Differential; BES, Basic Empathy Scale; JFE-HPS, Jefferson Scale of Empathy-Health Professions Students; IMTEE, Integrated Model of Training Evaluation and Effectiveness; KAOPS, Kogan’s Attitudes Toward Older People Scale; MSS, Maxwell Sullivan Scale; Palmore FAQ-1, Palmore Facts About Aging Quiz; UCLA-GA, UCLA Geriatric Attitudes Test; WCOP, Willingness To Care For Older People Scale; calculated effect size Cohen’s *d* (Lenhard and Lenhard 2017) variant 1 effect size represents pre-to-post differences in the IG; positive/negative effects reporting increased/decreased attitudes/empathy; calculated effect size Cohen’s *d* (Morris 2008) variant 3 represents post differences between IG-CG

^a^Mean effect size for the two attitude measures MSS and UCLA-GA

^b^Mean effect size for positive and negative KAOPS scores

**Table 3:** Results and calculated effect sizes of the included quantitative studies with non-controlled within-subject designs

Author(s)	Conducted questionnaire	T0 mean ± SD	T1 mean ± SD	T2 mean ± SD	T3 mean ± SD	T4 mean ± SD	*p* T0–T1	*p* T1–T2	*p* T0–T4	*d* T0–T1	Findings
Akpinar Söylemez (2021)	KAOPS	145.80 ± 19.64	153.19 ± 20.11				.001			0.27	↑Sig. increased score in attitudes
	Positive	72.34 ± 13.52	78.46 ± 12.75				.274			0.32	↑Increased score in pos. attitudes
	Negative	62.54 ± 11.98	61.27 ± 12.84				.001			- 0.01	
Allen (2018)	ASD	77.4 ± 18.4	75.6 ± 21.1				.36			- 0.09	No sig. difference in attitudes T0 to T1
Hsu et al. (2016)	Attitude Toward the Older People Scale	4.18 ± 0.61	4.27 ± 0.52				.57			0.16	No sig. change in attitudes toward older adults
	Willingness to Care	N = 92 (67.8%)	N = 105 (78.4%)				.001				↑ Increased willingness to care for older adults improved
Jeong et al. (2017)	Semantic Differential	3.92 ± 0.59	4.26 ± 0.49			3.73 ± 0.57	< .001	< .001	.022	- 0.63	↓ Attitudes decreased T0 to T1 ↑ Attitudes increased T0 to T4
Jeong and Kwon (2020)	Semantic Differential	4.00 ± 0.77	3.84 ± 0.64	3.97 ± 0.63	4.02 ± 0.59	4.08 ± 0.53	.010	.003	.003	- 0.23	↓ Attitudes become decreased T0 to T1
	Behavior toward elderly scale	3.41 ± 0.29	3.43 ± .0.30	3.41 ± .0.36	3.51 ± 0.33	3.54 ± 0.36			.002	0.07	↑ Attitudes become more positive after sharing their feelings
Losa Iglesias (2020)	JSE-HPS	86.59 ± 6.31	90.11 ± 6.83				.003			0.54	↑Sig. increased empathy
	TMMS-24	26.77 ± 6.33	28.51 ± 6.62				.014			0.27	
	PANAS	22.44 ± 6.38	20.88 ± 7.00				.030			- 0.23	
Perot et al. (2020)	Free association test^a^ Decreased vision Decreased hearing Loneliness	N of citing 272 265 32	N of citing 280 240 76				n/a	n/a			Participants’ free associations regarding difficulties experienced by older people were impacted by the ageing− simulation experience in various areas
Robinson and Rosher (2001)	ASD	124.35	116.14				n/a	n/a			↑ (Lower scores) increased attitudes toward older adults post simulation experience
Ross et al. (2013)	Confidence scale IMTEE	5.2 ± 0.88	6.0 ± 0.65				< .001			1.03	↑ Sig. increased confidence post intervention
Sari et al. (2020)	BES	70.8 ± 8.38	75.00 ± 11.36				.006			0.42	↑ Sig. increased score in empathy scale
	KAOPS	126.37 ± 9.21	135.33 ± 9.65				.001			0.95	↑ Sig. increased score in attitudes scale
Varkey et al. (2006)	MSS										
	Attitudes	28.19 ± 2.61	28.99 ± 2.14				n/a			0.34	↑ (not sig) increased in attitudes toward older adults
	Empathy	5.12 ± 1.40	4.60 ± 1.19								
	ASD	n/a	n/a				n/a			0.40	↑ Sig increased empathy

ASS, Age Simulation Suit; ASD, Aging Semantic Differential; BES, Basic Empathy Scale; JFE-HPS, Jefferson Scale of Empathy-Health Professions Students; IMTEE, Integrated Model of Training Evaluation and Effectiveness; KAOPS, Kogan’s Attitudes Toward Older People Scale; MSS, Maxwell Sullivan Scale; Palmore FAQ-1, Palmore Facts About Aging Quiz; UCLA-GA, UCLA Geriatric Attitudes Test; WCOP, Willingness To Care For Older People Scale; calculated effect size Cohen’s *d* (Lenhard and Lenhard 2017) variant 1 effect size represents pre-to-post differences in the IG; positive/negative effects reporting increased/decreased attitudes/empathy

^a^Perot et al. (2020) results were not fully listed due to the missing comparability of used questionnaire

The predominant purpose of these studies was to investigate the usefulness of ASS to improve empathy and/or attitudes toward older adults and/or raise the awareness regarding challenges of the ageing process among samples of younger adults. The most frequent outcome measures were attitudes (*n* = 12), followed by assessments of empathy and understanding (*n* = 9), willingness to care for (*n* = 2) or behavior toward older adults (*n* = 1). As different scales vary in their coding procedures (i.e., lower scores in ASD, SD or MSS indicate more positive attitudes toward older adults), the term *increased* is used in the following to indicate more positive and the term *decreased* is used to indicate more negative attitudes or empathy. Hence, positive effect sizes (Cohen’s *d*) represent an improvement within the respective construct.

Regarding the 12 studies that assessed *attitudes* toward older adults, our effect size calculations (pre-to-post) with the reported scores of the two randomized controlled trials indicated small (*d* = 0.36) and medium-sized positive effects (*d* = 0.71). Our calculations on quantitative non-randomized studies (*n* = 8) revealed small positive effects (*n* = 3; range: *d* = 0.34-.46), one large positive effect (*n* = 1; *d* = 4.43), two small negative effect sizes (*n* = 2; *d* =  - 0.23 and  - 0.36), and no effect (*n* = 2; *d* =  - 0.09 and *d* = 0.16), respectively. For the quantitative parts of the two mixed method studies, our calculations indicated one large positive (*d* = 0.95) and one medium negative (*d* = − 0.63) effect. Of note, two studies that initially found negative effects on attitude measures after the ASS intervention reported positive changes in a later follow-up (Jeong et al. 2017; Jeong and Kwon 2020). Overall, the weighted mean effect size for pre-to-post changes in attitudes was *d* = 0.33, corresponding to a small effect; detailed results for each study can be found in Tables 2 and 3. We additionally calculated effect sizes between groups for the five studies that used controlled designs (IG vs CG, see Table 2). The weighted mean effect size for attitudes in those between-subjects designs was *d* = 0.29, corresponding to a small effect.

**Table 4:** Results and calculated effect sizes of the included quantitative studies with physical performance measurements

Author(s)	Assessments	Without ASS	With ASS	*p*	*d*	Findings
Lavallière et al. (2017)	Eyes open (s)	29.69 ± 1.08	28.63 ± 2.40	n/a	- 0.57	
	Eyes closed (s)	18.63 ± 8.05	11.30 ± 6.69	< .001	- 0.99	
	Shoulder abduction (°)	169.2 ± 15.7	156.5 ± 20.0	< .001	- 0.71	↓ Trend to decreased performance in neck and shoulder range of motion as well as hamstring flexibility
	Cervical extension (°)	83.0 ± 12.0	69.5 ± 15.0	< .001	- 0.99	
	Lateral neck flexion (°)	39.0 ± 8.5	27.7 ± 9.0	< .001	- 1.29	
	Flexibility (cm)	25.84 ± 13.54	21.79 ± 12.5	< .001	- 0.31	
	Gait seconds	7.13 ± 0.84	7.87 ± 1.22	< .01	- 1.66	↓ Trend to decreased performance gait parameters
	Velocity (m/s)	1.42 ± 0.17	1.31 ± 0.024	< .01	- 1.51	
	Number of steps within 10 m	14.25 ± 1.38	15.09 ± 1.69	< .01	- 1.36	
Scherf (2014)	Subjective physical load	g: 2.80 ± 1.03	g: 6.7 ± 2.21	.000	- 2.26	↓ Subjective physical load increased when working with the ASS
		y: 4.33 ± 2.74	y: 6.33 ± 2.55	.000	- 0.76	
		r: 1.56 ± 1.33	r: 7.00 ± 2.12	.000	- 3.07	
	Time to complete the task (s)	g: 191.65 ± 29.16	g: 157.70 ± 25.05	.000	- 1.25	↓ Sig. increased time to complete the task
		y: 199.17 ± 32.04	y: 160.88 ± 24.86	.000	- 1.34	
		r: 214.94 ± 30.79	r: 154.67 ± 22.71	.000	- 2.23	
	Heartrate (heartbeats/min)	g: 117.00 ± 17.16	g: 102.44 ± 15.66	.000	- 0.89	↓ Sig. increased heart rate while working
		y: 121.44 ± 18.85	y: 106.78 ± 14.85	.000	- 0.86	
		r: 128.44 ± 19.62	r: 107.78 ± 10.91	.002	- 1.30	
Vieweg and Schaefer (2020)		Female / male	Female / male			
	Leg strength (n)	27.0 ± 4.0 / 27.6 ± 4.9	21.7 ± 3.3 / 23.7 ± 4.1	.001	- 1.45	↓ Sig. reduced leg strength
	Arm strength (n)	19.7 ± 2.9 / 23.5 ± 4.3	16.6 ± 3.5 / 20.6 ± 3.9	.001	-0.96	↓ Sig. reduced arm strength
	Aerobic endurance (n)	122.4 ± 12.4 / 130.5 ± 13.4	93.5 ± 13.5 / 107.5 ± 10.5	.001	- 2.23	↓ Sig. reduced aerobic endurance
	Hip flexibility (cm)	25.7 ± 6.5 / 17.8 ± 10.3	21.2 ± 5.4 / 14.8 ± 12.3	.001	- 0.75	↓ Sig. reduced hip flexibility
	Shoulder flexibility(cm)	7.1 ± 5.5 / 2.8 ± 9.3	- 3.3 ± 6.5 / −10.4 ± 12.0	.001	- 1.73	↓ Sig. reduced shoulder flexibility
	TUG (s)	3.6 ± 0.6 / 3.3 ± 0.3	4.4 ± 0.4 / 4.1 ± 0.5	.001	- 1.57	↓ Sig. reduced functional mobility TUG
	Dominant hand	17.3 ± 1.47 / 15.3 ± 1.85	15.0 ± 1.56 / 14.4 ± 2.15	< .001	- 0.86	↓ Sig. decreased performance in PPT
	Non − dominant hand	15.8 ± 2.01 / 14.6 ± 1.25	14.4 ± 1.69 / 13.9 ± 2.19	< .001	- 0.71	↓ Sig. decreased performance
	Both hands	13.9 ± 1.40 / 11.8 ± 1.17	12.1 ± 1.30 / 11.9 ± 2.22	< .001	- 1.91	↓ Sig. decreased performance
	Assembly	39.4 ± 6.69 / 34.7 ± 6.86	34.6 ± 8.21 / 33.8 ± 9.66	< .001	- 0.26	↓ Sig. decreased performance
	Shirt − buttoning (n buttons)	25.00 ± 4.85	9.00 ± 4.06	< .001	- 1.23	↓ Sig. decreased performance
	Digital Symbol Test	n/a	n/a	.01	- 2.26	↓ Sig. decreased performance
Watkins et al. (2021)	Functional reach (in cm)	36.90 ± 2.40	31.25 ± 10.1	< .005	- 0.77	↓ Sig. decreased performance
	Timed Up and Go (in s)	6.68 ± 0.63	8.41 ± 0.79	< .005	- 2.42	↓ Sig. increased time to complete TUG
	Berg Balance Scale (score)	56 ± 0	55 ± 2	.01	- 0.71	↓ Sig. decreased performance
Zijlstra et al. (2016)	Route efficiency	0.76 ± 0.75	0.84 ± 0.84	.361	0.10	Tendency but no sig. decreased route efficacy
	Walking speed	3.44 ± 1.28	2.78 ± 0.15	< .001	- 0.72	↓ Sig. decreased walking speed with ASS
	Heart rate	110.33 ± 33.34	124.64 ± 36.80	< .001	- 0.41	↑ Sig. increased heartrate
	Respiratory rate	− 8.69 ± 15.92	− 4.80 ± 1.04	< .001	- 0.35	↑ Sig. increased respiratory rate

ASS, Age Simulation Suit; n, numbers, s, seconds, cm, centimeters, °, degrees, min, Minute; calculated effect size Cohen’s *d* (Lenhard and Lenhard 2017) variant 1 effect size represents pre-to-post differences; positive/negative effects reporting increased/decreased performance, Lauenroth et al. (2017) was excluded from the table due to not conducting a within-subjects design and therefore missing comparability

Regarding the outcomes concerned with *empathy* for older adults, our effect size calculations indicated no effect *(n* = 1; *d* = 0.12) for the randomized trial within the mixed methods design, small and medium effects (*n* = 3; *d* = 0.40, *d* = 0.48, *d* = 0.54) for the non-randomized quantitative designs, and one small and one large effect for the quantitative parts of the two mixed method studies (*n* = 2, *d* = 0.42 and *d* = 1.03). Two studies reported no adequate data to compute effect sizes. The weighted mean effect size from pre-to-post changes in empathy was *d* = 0.54, corresponding to a medium-sized effect. We additionally calculated effect sizes between groups for empathy (IG vs. CG, see Table 2) for the three studies that used controlled designs. The weighted mean effect size in those between-subjects designs was *d* = 0.07, corresponding to no meaningful effect.

From the six qualitative and mixed methods studies, four conducted semi-structured interviews or discussions, where participants could share their experiences after wearing an ASS (Bowden et al. 2020; Jeong et al. 2017; Ross et al. 2013; Sari et al. 2020). In their analysis of focus groups, Bowden et al. (2020) reported enhanced insight for the process of ageing among the participants and growing empathy for their future self. Jeong et al. (2017) used in-depth interviews and results indicated a better understanding for challenges due to physical and sensory impairments. Reports on subjectively increased empathy and the feeling of having gained a better understanding of the process of ageing were communicated across all ASS studies evaluating qualitative data. Beyond that, Jeong et al. (2017) reported subjectively increased willingness to care for older adults, Sari et al. (2020) reported higher awareness regarding difficulties with activities of daily living, and Ross et al. (2013) reported better understanding for specific needs of older people, i.e., fear of falling and feeling safe. Lavallière et al. (2017) reported that the participants rather attributed perceived difficulties to complete given tasks to environmental restrictions than to the ASS, i.e., narrow aisles in a supermarket. Therefore, the analysis did not indicate differentiated awareness of age-related limitations caused by the suit. Lee and Teh (2020) included a practical interaction with older adults in their polypharmacy workshop before the ASS intervention. Afterward, they used open-ended questionnaires and identified three themes (1) “lending an ear”, which meant taking more time to listen, (2) “sense of respect” meaning realizing the challenges in the lives of older adults, and (3) “understanding the emotion,” which indicated the importance of empathy in healthcare.

#### Physical outcomes

Six studies assessed the effects of ASS on physical performance (see Table 4). Four studies used validated (geriatric) assessments (Lauenroth et al. 2017; Lavallière et al. 2017; Vieweg and Schaefer 2020; Watkins et al. 2021), two focused on self-developed or modified established tests (Scherf 2014; Zijlstra et al. 2016). We calculated Cohen’s *d* effect sizes for the differences with and without the ASS, indicating within-subject or pre-to-post differences (Lenhard and Lenhard 2017). Negative effect sizes represent decreased physical performance in respective tasks, abilities, or physiological parameters. Lavallière et al. (2017) found significantly decreased performance while wearing an ASS in postural balance tests (standing on both legs with eyes open: *d* =  -0.57; eyes closed: *d* =  -0.99), flexibility tests, range of motion (shoulder, neck, cervical spine; range from *d* =  -0.71 to  -1.29), and parameters indicating altered gait (number of steps: *d* =  - 1.51, duration: *d* =  - 1.66, and velocity: *d* =  -1.36) on a four-meter walkway. Watkins et al. (2021) conducted similar comparisons with and without ASS and reported significantly decreased performances in the Functional Reach Test (FRT; *d* =  -0.77), Timed Up and Go (TUG; *d* =  - 2.42), and Berg Balance Scale (BBS; *d* =  -0.71). Vieweg and Schaefer (2020) assessed the Functional Fitness Test (FFT) and Perdue Pegboard Test (PPT) and reported decreased performance when wearing the ASS (physical performance: range from *d* =  -0.71 to -2.23, fine motor tasks: range from *d* =  -0.42 to  - 2.17). They also included a cognitive task conducted before and while wearing an ASS. Results of the Digit Symbol Test, an indicator of information processing speed, demonstrated an increased time to perform the task with the ASS, indicating a pronounced decline in this cognitive domain (*d* =  -1.77).

**Table 5:** Scores with age simulation suit compared to reference values for physical performance assessments

Reference values per age groups; female/male					
Assessment	Scores with ASS mean ± SD (age range in years) Female/male	50–59 years	60–69 years	70–79 years	80–99 years
Velocity^a^ (cm/s)	120 ± 19.9 (18–29)				
Lauenroth et al.(2017)	128 ± 23.3 (30–39) 133 ± 27.5 (40–49) 124 ± 18.8 (50–59)	**131.3/**143.3	**124.1/133.9**	116 ± 20/117 ± 16	112 ± 17/122 ± 15
Zijlstra et al. (2016)	85.66 ± 35.56				
Lavalliere et al. (2017)	131 ± 24				

	Scores with ASS	50–59 years	60–64 years	65–69 years	70–74 years	75–79 years	80–84 years	85–89 years
Step length^b^ (cm) Lauenroth et al. (2017)	69.7 ± 8.08 (18–29) 72.9 ± 10.3 (30–39) 73.2 ± 8.19 (40–49) 69.1 ± 6.96 (50–59)	n/a	n/a	n/a	61 ± 9/69 ± 8	59 ± 7/68 ± 7	55 ± 7/65 ± 8	54 ± 9/59 ± 10
Step time^a^ (sec) Lauenroth et al. (2017)	0.55 ± 0.05 (18–29) 0.55 ± 0.04 (30–39) 0.53 ± 0.04 (40–49) 0.56 ± 0.06 (50–59)	n/a	n/a	n/a	0.68 ± 0.10/0.75 ± 0.07	0.67 ± 0.08/0.72 ± 0.06	0.71 ± .07/0.74 ± .06	0.72 ± 0.09/0.78 ± 0.11
*FFTc f/m* Vieweg et al. (2020)								
Leg strength (n)	21.7 ± 3.3/23.7 ± 4.1	n/a	14.5 ± 4.0/16.4 ± 4.3	13.5 ± 3.5/15.2 ± 4.5	12.9 ± 3.6/14.5 ± 4.2	12.5 ± 3.8/14.0 ± 4.3	11.3 ± 4.2/12.4 ± 3.9	10.3 ± 4.0/11.1 ± 4.6
Arm strength (n)	16.6 ± 3.5/20.6 ± 3.9		**16.1 ± 4.6**/**19.0 ± 4.7**	15.2 ± 4.3/18.4 ± 5.3	14.5 ± 4.4/17.4 ± 5.0	14.0 ± 4.4/16.2 ± 4.6	13.0 ± 4.1/16.0 ± 4.3	12.2 ± 3.8/13.6 ± 4.3
Aerobic endurance (n)	93.5 ± 13.5/107.5 ± 10.5		**91 ± 24/101 ± 21**	90 ± 26/101 ± 23	84 ± 25/95 ± 23	84 ± 24/91 ± 27	75 ± 23/87 ± 24	70 ± 22/75 ± 24
Hip flexibility (cm)	21.2 ± 5.4/14.8 ± 12.3		5.3 ± 10.2/1.5 ± 12.2	5.1 ± 9.1/0.0 ± 11.7	3.6 ± 9.4/-1.0 ± 11.7	- 5.4 ± 10.4/- 2.8 ± 11.9	1.3 ± 9.4/-5.1 ± 12.7	- 0.3 ± 9.4/- 6.1 ± 10.4
Shoulder flexibility(cm)	- 3.3 ± 6.5/- 10.4 ± 12.0		- 1.8 ± 8.9/- 8.6 ± 12.2	**- 3.0 ± 9.4**/**- 10.4 ± 12.4**	- 4.3 ± 9.7/- 11.4 ± 12.4	- 5.3 ± 10.4/- 14.2 ± 13.0	- 6.6 ± 10.7/- 14.5 ± 13.7	- 9.9 ± 11.4/- 15.7 ± 12.2
Timed Up and Go (s)	4.4 ± 0.4/4.1 ± 0.5		5.2 ± 1.2/4.7 ± 1.3	5.6 ± 1.2/5.1 ± 1.2	6.0 ± 1.6/5.3 ± 1.3	6.3 ± 1.6/5.9 ± 1.9	7.2 ± 2.2/6.4 ± 1.8	7.9 ± 2.5/7.2 ± 2.6

	Scores with ASS	50–59 years	60–69 years		70–79 years		80–89 years	
*PPT*^*d*^* f/m *Vieweg et al. (2020)								
Dominant hand (n)	12.8 ± 0.95/12.2 ± 1.90	15.0 ± 1.56/14.4 ± 2.15	14.6 ± 2.03/13.6 ± 1.74		13.8 ± 1.27/**13.0 ± 1.90**		**12.9 ± 1.80**/10.8 ± 1.33	
Non-dominant hand (n)	12.0 ± 1.67/11.5 ± 1.36	14.4 ± 1.69/13.9 ± 2.19	13.9 ± 1.78/13.1 ± 1.56		**12.9 ± 1.52**/**12.4 ± 1.48**		11.3 ± 2.05/10.6 ± 1.84	
Both hands (n of pairs)	9.8 ± 1.81/9.1 ± 1.28	12.1 ± 1.30/11.9 ± 2.22	11.6 ± 1.87/10.9 ± 1.46		**10.5 ± 1.19**/10.4 ± 1.27		9.2 ± 1.92/**8.5 ± 1.21**	
Assembly (n)	25.8 ± 7.61/24.3 ± 5.51	34.6 ± 8.21/33.8 ± 9.66	31.7 ± 6.83/28.0 ± 5.06		29.1 ± 4.85/27.5 ± 5.06		**21.9 ± 4.54**/**21.5 ± 4.81**	
Eyes open (s) Lavalliere et al. (2017)	28.63 ± 2.40	n/a	n/a		n/a		n/a	
Eyes closed (s)^e^Lavalliere et al. (2017)	11.30 ± 6.69	22.9/23.2	18.3/19.7		13.2/15.4		**9.1/9.0**	
TUG^f^ (s) Watkins et al. (2021)	8.41 ± 1.73	n/a	7.91		**8.67**		11.68	
BBS^g^ (score) Watkins et al. (2021)	55 ± 0.5	n/a	**55 ± 2/55 ± 1**		53 ± 4/54 ± 3		50 ± 3/53 ± 2	

	Scores with ASS	41–69 years	70–87 years
FRT (cm)^h^ Watkins et al. (2021)	31.25 ± 5.8	35.1 ± 5.6/37.8 ± 5.6	26.7 ± 8.9/**33.5 ± 4.1**

cm = centimeter, s = seconds; leg strength sit to stand (*n* = number of full stands), hip/shoulder flexibility (in cm), arm strength (*n* = number of bicep curls), aerobic endurance (*n* = number of steps within 2 min) ASS = Age simulation suit; BBS = Berg balance scale; FFT = Functional fitness test; FRT = Functional reach test; PPG = Perdue pegboard test; TUG = Timed up and go test; values in bold correspond to reference values of the certain age range of older adults

^a^Bohannon and Williams Andrews (2011)

^b^Hollman et al. (2011)

^c^Rikli and Jones (1999)

^d^Agnew et al. (1988)

^e^Agrawal et al. (2011)

^f^Long et al. (2020)

^g^Steffen et al. (2002)

^h^Duncan et al. (1990)

The two remaining studies used additional physiological and subjective indicators to quantify physical load. Scherf (2014) monitored younger assembly line workers accomplishing a task (putting together automotive parts) with and without an ASS and additionally compared them with older employees without an ASS. In comparison with measures without ASS, participants’ heart rate (*d* =  -1.02), subjective physical load (*d* =  -2.03), and completion time (*d* =  -1.02) increased, which characterized a decreased performance. Zijlstra et al. (2016) assessed heart- and respiratory rate, route efficiency, and walking speed in a wayfinding task in a hospital. Findings indicated that while wearing an ASS, participants had a higher heart rate (*d*  - 0.60) and respiratory rate (*d* =  -0.35), and were walking significantly slower (*d* =  -0.72); no significant changes were found in route efficiency (*d* =  -0.15).

### Findings on research question 2: validity of age simulation suits regarding various age-related impairments

Five of the six studies on physical performance measures provided data that could be used for our second aim, namely to clarify if ASS are valid in terms of a realistic simulation of normative age-related performance decreases (see Table 5). To classify and compare study results, we used established reference values, if available.

We identified five studies comparing ASS physical performance data with reference data. Lauenroth et al. (2017) examined various gait variables (velocity, step length, step time, base width) and compared them between different age groups. Four younger groups (18-29, 30-39, 40-49, and 50-59 years) conducted the assessment with the ASS, the older participants (60-69, 70-85 years) without the ASS only. Results demonstrated that step length and velocity were comparable between participants aged 40-49 years with ASS and those aged 60-69 years without ASS in the study (respectively, step length for 50-59 years with ASS was comparable to 70-85 years without ASS). The results of participants aged 40-49 years with ASS for gait velocity were also comparable to external reference values for females aged 60-69 years, but not for male reference values. Younger participants, aged 18-29 years, wearing ASS were slightly faster than reference values for 70-79 year old males and females. For step length and step time, participants’ results with ASS were still better than reference values of adults older than 70 years. Established reference values for base-width were not available.

Lavallière et al. (2017) assessed different physical outcomes, but without relating these to available reference values. Their reported gait velocity of younger adults (20-29 years) with ASS, conducted on a ten meter walkway, corresponded to reference values for females aged 50-59 and males aged 60-69 years (Bohannon and Williams Andrews 2011).

In the study of Zijlstra et al. (2016), gait velocity was calculated by the time to complete a wayfinding task and the measured distance walked when wearing an ASS (participants’ age: 20.0 ± 1.8 years). Reported results were still better than reference values of adults aged 50 years and did not correspond to the target group of older adults of 65 years and older (Bohannon and Williams Andrews 2011).

Vieweg and Schaefer (2020) conducted the FFT with a group of students (20-28 years) and compared their results with reference values from Rikli and Jones (1999). The included arm strength test revealed results comparable to reference values of adults aged 60-64 years. Participants’ leg strength also decreased when wearing the ASS. Nevertheless, men still did better than reference values for people in their mid-50 s. Results of the TUG indicated a decline with ASS that was comparable to 60-64 years old adults, which was similar for aerobic endurance (2 min stepping test). Hip flexibility with ASS was still better than normative values of adults 60-64 years and shoulder flexibility was comparable to 65-69 years old adults (male/female).

Finally, Watkins et al. (2021) noted that three of their thirty participating students (20-40 years) were not comparable to reference values of middle aged adults (due to still very high performance), though conducting the FRT, six students wearing the ASS reached normative values of 41-69 years old adults, while 21 students reached values of 70-87 years old adults (Long et al. 2020). For the TUG, they reported longer completion time with ASS for all participants. Thirteen participants met normative values for 60-69 years, five for 70-79 years and one for 80-89 years old adults.

Taken all five studies together, results indicated that ASS reduced the physical performance in almost all domains, but overall not to the extent that participants were comparable to older adults’ reference values, when wearing the ASS.

## Discussion

The primary purpose of this review was to synthesize the current research on ASS and their effects on psychological and physical performance outcomes. Second, the validity of ASS in terms of a realistic simulation of the normative ageing process particularly in its functional domains has been a target of the paper. 26 studies with publication years ranging from 2001 to 2021 were finally included, of which twenty addressed psychological outcomes such as empathy for and attitudes toward older adults, while six focused on physical assessments. Seventeen of the included studies were published in the last 5 years, thus demonstrating that research on ASS found much interest recently. Only five articles contained information that allowed an estimation of the age validity of wearing an ASS, i.e., by providing data from established assessments we could compare to reference values of older adults.

### Effects of age simulation suits: psychological outcomes

The majority of studies reported a positive effect on empathy for and attitudes toward older adults. For all studies assessing pre-to-post changes, the weighted mean effect size was *d* = 0.33 for attitudes and *d* = 0.54 for empathy. However, some of the rare studies that used controlled designs did not find meaningful differences between the control group and ASS group (Cheng et al. 2020; Lee and Teh 2020), or even negative effects on attitudes immediately after wearing an ASS (Jeong et al. 2017; Jeong and Kwon 2020; Lucchetti et al. 2017). In conclusion, the effects of wearing an ASS on psychological outcomes seem to be overall positive; still, the rather short time frames covered have to be considered. That is, only three studies assessed outcomes in follow-ups longer than three weeks (Jeong et al. 2017; Jeong and Kwon 2020; Lee and Teh 2020).

Taking a more critical look, some of the positive effects cannot be solely attributed to the ASS interventions, as similar results in control groups led to the conclusion that addressing the feeling of being older could be sufficient to improve attitudes toward older adults. As one study indicated that “placebo clothes” caused similar reactions the mind-set of being older might have influenced participants in the same way (Cheng et al. 2020).

Some articles reported reduced positive attitudes toward older adults or decreased empathy immediately after the ASS intervention. The authors concluded that the simulation raised negative emotions, such as anxiety and fear of future physical or sensory limitations, which might lead to these effects. This finding underlines the importance of providing the opportunity to reflect on the experiences. Furthermore, the measurements largely focused on attitudes and empathy, whereas multifaceted views on one’s own ageing process such as awareness of age-related gains and losses (Diehl and Wahl 2010) or ageing-related changes in stereotypes in diverse domains (Kornadt and Rothermund 2011) have not been studied yet. Similarly, research has not addressed how ASS affect broader constructs related to more general views on ageing, i.e., age stereotypes in different life domains, perceived obsolescence, or health-related risk perception.

Some of the rather descriptive designs or qualitative evaluations gave the impression of not being a priori planned as a study, but rather as a post-hoc course evaluation. This may have led to a publication bias, with positive effects being more likely to be published, whereas mixed or negative results might be underrepresented. In addition, the often missing randomization and blinding of assessors, as well as the assessment of psychological outcomes prone to social desirability may have resulted in biased results. Qualitative results might be biased even more by social desirability, i.e., answering in a manner that will be viewed favorably by other students or the investigator in focus groups. However, the setting and expectation of improvements are rather obvious in most designs. In summary, the limited number of controlled studies only allows for cautious and preliminary conclusions and further research is needed.

### Effects of age simulation suits: physical outcomes

Six included studies focused on a variety of performance-based measures addressing the areas of gait parameters (*n* = 3), flexibility (*n* = 3), functional mobility (*n* = 2), balance (*n* = 2), physiological changes (*n* = 2), strength (*n* = 1) and aerobic endurance (*n* = 1). Strongest decreases in terms of effect sizes due to wearing an ASS were found for flexibility and functional assessments, whereas smaller decreases appeared in balance tests. In most studies, established assessments such as the TUG, FFT, and gait performance were used (*n* = 5). Limitations with respect to accuracy (i.e., velocity measured with stopwatches) could be overcome with more advanced technical systems. Moreover, covariates such as participants’ fitness level or physical activity habits should have been taken into account.

For future ASS studies focusing on physical performance, more complex tasks, more diverse established assessments and everyday activities might have the potential to depict age-related limitations that are often multidimensional and might not be replicated in isolated measurements. For example, motor-cognitive dual tasks, dynamic balance, or (instrumental) activities of daily living could be considered.

### Validity of ASS in terms of simulating the ageing experience realistically

For our second objective, to summarize and quantify indicators that can be used for estimations of validity, we were able to draw upon findings from five studies, with three studies assessing gait velocity. The consideration of gait variabilities offers a well-established quantification in locomotion, bearing the advantage that reference values are available for many parameters. Results indicated a decreased performance for young and middle-aged participants and resulted in an “instant ageing” effect of about 20-40 years, when comparing established gait assessments to reference values. The extremely reduced gait velocity in one study (Zijlstra et al. 2016) was not representative for older adults. Though, it should be considered that the authors calculated gait velocity after completing a full wayfinding task, whereas reference values are mostly lab-based data with known limitations, but without distractions. However, reported step length and step time did not reach the levels of older reference groups and the participants still demonstrated better performance (Lauenroth et al. 2017). Overall, results indicated that performance scores of the assessments with ASS were often not corresponding to age norms of adults aged 60-64 years or older, but still resembled younger age groups i.e., in leg and arm strength or aerobic endurance. One explanation might be a general good fitness level of participants, which may not be representative. Moreover, length of habituation phase and length of simulation intervention can influence physical performance and has to be considered. Still, complex assessments (TUG, BBS) demonstrated that more than 50% of participants had an increased risk of falling while wearing the ASS and that scores resulted in an “instant ageing” of about 30-40 years. These test are known as the gold standard for evaluating balance limitations in older adults, as impaired balance is one of the major risks for falls in older adults and therefore an important indicator for a typical ageing process (Ambrose et al. 2013). Regarding flexibility measurements, the three respective studies reported mixed findings. Some isolated flexibility measurements seemed to be overstated (e.g., neck), while others were in line with reference values of older adults (FFT shoulder and FRT overall score). One study assessed the Digit Symbol Test with and without an ASS and found that the performance with ASS was comparable to reference values of adults older than eighty. However, the authors assumed that a large portion of the decline was due to visual impairments rather than cognitive challenges.

In conclusion, physical performance decreases could be simulated among younger and middle-aged participants in most assessments, but predominantly not to the extent that represents adults older than 65 years or even fourth age (80+). Some of the suppliers of ASS specify certain age ranges (i.e., mid-70 s; AGNES ASS) that should be reached with their ASS or claim that users age 30 to 40 years (i.e., GERT ASS), but those assumptions have not been verified with data yet. Our review provides first insights but points out the need for differentiation regarding the population under study with the ASS and the specific tests that are applied. The mentioned studies reinforced the attempt to use of ASS to mimic typical age-related impairments, but should be recognized as a start or proof of concept.

### Strengths and limitations

This review’s focus on rather homogeneous ASS interventions, thus excluding ageing and geriatric games, which are conducted with people only observing, giving not all participants the chance to experience the simulation, and the consideration of a broad range of outcomes can be seen as strengths and a new approach to the matter. While earlier reviews focused on psychological outcomes only (Bowden et al. 2021; Coelho et al. 2017; Eost-Telling et al. 2020), we extended the synthesis regarding performance-based assessments in our first objective, calculated effect-sizes wherever possible and provided insights on validity estimates in our second objective. Limitations included quite large variations in method quality and study designs and little to no information and consideration of confounders (i.e., sociodemographic information, health status, previous experiences with older adults) in the included studies, which may reduce the reliability of results. This adds on to a possible publication bias resulting in the under-representation of negative results. As only studies in English or German language were included, and samples were predominantly drawn from Western, educated and industrialized populations, the generalizability of findings is also limited.

### Conclusion

ASS play a prominent role in various contexts as an educational device able to evoke empathy and better understanding of what it means to get older. Considering this, it would be highly desirable to be able to rely on robust research supporting that ASS devices are able to fulfil both, enhancing empathy and positive views on ageing as well as doing this based on a realistic and valid simulation of the typical ageing process. Regarding the rapid growth of research on ASS interventions with the large majority of the included studies published in the recent 5 years, there indeed seems to be a promising development in this research area. Largely consistent with earlier reviews focusing on psychological outcomes of wearing an ASS, predominantly positive effects on attitudes and empathy toward older adults were identified, although effect sizes were not calculated in earlier reviews and showed large variation in our work. The existing research reporting in some instances conflicting findings, sometimes pointing in a more negative direction of ASS effects, unfortunately does not allow for definite conclusions under which conditions such negative consequences are likely to occur. This would be an important task for future research. Given that the awareness of ageing processes and the ability to change perspectives are important soft skills for health care professions. Given that the simulation of older age might help younger adults such as those in midlife to better prepare for their own ageing, ASS indeed seem to be an important resource for future ageing societies on different levels. Regarding a range of key physical outcomes important for independent functioning in everyday life, large effects were identified, although this part of the previous research is still relatively small. Therefore, research on a diversity of outcomes echoing everyday challenges including more complex everyday tasks such as doing chores or cooking would be an important addition. Still, the crucial point is to simulate a range of motor-related everyday tasks in a realistic and age-valid way. Here, considering the domains of gait, functional mobility and strength, only limited evidence is available for the accurate simulation of 65+ years older adults with younger participants wearing an ASS. Future research should follow robust (controlled) research designs, include follow-up measurements, and reduce the likelihood of social desirability, e.g., by using less obvious questions and drawing on anonymous questionnaires instead of “open” data collection methods in seminars. The diversity of study populations should be considered to a larger extent, in particular in terms of age range. For example, it would be important to know, considering the general population, whether the effects of wearing an ASS are different for those in early adulthood versus those in midlife versus those in young-old age. Further, as at least some adverse effects of wearing an ASS were observed, it seems appropriate to recommend that the ASS should only be used in combination with gerontological expert supervision able to provide a comprehensive and differentiated picture of the ageing process. Finally, more evidence supporting the validity of the age simulations by an ASS might help rehab scientists and engineers who want to use ASS in the creation and improvement of technical devices for older adults. That is, the ASS may in the long run serve ageing societies on multiple levels, if additional research proves its usefulness.

## Supplementary Information

Below is the link to the electronic supplementary material.

Supplementary file1 (DOCX 15 KB)

## Data Availability

The data that support the findings of this study are available from the corresponding author upon request.
