# Supplementary material for: Effects of age simulation suits on psychological and physical outcomes: a systematic review
Source: Eur J Ageing. 2022 Sep 22;19(4):953–76. doi: 10.1007/s10433-022-00722-1 (PMC9729636; doi:10.1007/s10433-022-00722-1)
Supplement: Supplementary file 1 — Supplementary file1 (DOCX 15 KB) [file 10433_2022_722_MOESM1_ESM.docx]

**Supplementary material**

Table 1: Search terms and initial results per database

| Database | Search terms | results (n) |
| --- | --- | --- |
| Pubmed  Web of Science  Google Scholar | ("Age suit*" OR "Age simulation suit*" OR "Age simulation*" OR "Ageing simulation*" OR "Aged simulation suit*" OR "Physical limitation suit*" OR "Senior simulation suit*" OR "Simulation of elderly" OR "Aging-related simulation*" OR "Aging game simulation*" OR "Simulation of aging*" OR "Elderly simulation*" OR "Gerontologic suit*" OR "Geriatric simulation" OR "Geriatric simulator") AND ("Age stereotype*" OR "Aging stereotype*" OR "Ageing stereotype*" OR "Image* of aging" OR "Image* of age" OR "Image* of ageing*" OR "Attitude*" OR "empathy" OR "Empathetic attitude*" OR "Empathic*" OR "Knowledge about older adults" OR "Understanding" OR "Learning experience" OR "Subjective age" OR ("efficacy" AND ("age" OR "aging")) OR ("evaluating” AND (“age” OR “aging”)) OR ("Evaluation" AND ("age" OR "aging")) OR ("Validation" AND ("age" OR "aging")) OR ("validity" AND ("age" OR "aging")) OR "sensitizing" OR "willingness to care" OR "product design" OR ("perception" AND ("older adult*" OR "age*")) OR "age-related change" OR "views on aging" OR "awareness of aging" OR "aging experience" OR "felt age" OR "self-perception of aging" OR "Mobility assessment" OR "Geriatric assessment*" OR "TUG" OR "Up-and-Go" OR "Timed-Up-and-Go" OR "Timed Up and Go" OR "SPPB" OR "Short Physical Performance Battery" OR "BBS" OR "Berg Balance Scale" OR "SFT" OR "Senior Fitness Test" OR "FRT" OR "Functional Reach Test" OR "grip strength" OR "gait" OR "gait analysis" OR "walking speed" OR "balance" OR “assessment*”)  Pubmed: AND "Aging/psychology"[Mesh] OR "Aging/physiology"[Mesh] OR "Geriatrics/education"[Mesh] OR "Geriatrics/psychology"[Mesh] OR “Attitude of Health Personnel*”[Mesh] OR "Problem-Based Learning"[Mesh] OR “simulation training”[Mesh] -> 956 | 1093  35  325 |
| Cinhal  PsychINFO  ProQuest  BASE  Cochrane | ("Age suit*" OR "Age simulation suit*" OR "Age simulation*" OR "Ageing simulation*" OR "Aged simulation suit*" OR "Physical limitation suit*" OR "Senior simulation suit*" OR "Simulation of elderly" OR "Aging-related simulation*" OR "Aging game simulation*" OR "Simulation of aging*" OR "Elderly simulation*" OR "Gerontologic suit*" OR "Geriatric simulation" OR "Geriatric simulator") | 32  25  284  116  9 |
| Reference search |  | 29 |
